# TIMP-2 inhibits metastasis and predicts prognosis of colorectal cancer via regulating MMP-9

**DOI:** 10.1080/19336918.2019.1639303

**Published:** 2019-07-11

**Authors:** Weimin Wang, Dan Li, Liangliang Xiang, Mengying Lv, Li Tao, Tengyang Ni, Jianliang Deng, Xiancheng Gu, Sunagawa Masatara, Yanqing Liu, Yan Zhou

**Affiliations:** a Institute of Traslational Medicine, Medical College, Yangzhou University, Yangzhou, PR China; b The Key Laboratory of Syndrome Differentiation and Treatment of Gastric Cancer of the State Administration of Traditional Chinese Medicine, Yangzhou, PR China; c Department of Oncology, Yixing Hospital Affiliated to Medical College of Yangzhou University, Yixing, Jiangsu, PR China; d Department of Physiology, School of Medicine, Showa University, Tokyo, Japan

**Keywords:** CRC, TIMP-2, MMP-9, prognosis, metastasis

## Abstract

Colorectal cancer has a common cause of morbidity and mortality. Therefore, it is urgent to detect reliable biomarkers to predict prognosis in CRC. Here, we determined the expression of TIMP-2 and MMP-9 in a  CRC tissue microarray by immunohistochemistry. We found that lower TIMP-2 or/and higher MMP-9 expression in cancer tissues was correlated with poorer overall survival (OS). TIMP-2 or MMP-9 expression was independent prognostic factors for CRC. Furthermore, TIMP-2 and MMP-9 expression had a synergistic role as efficient prognostic indicators for CRC patients. In vitro and in vivo, TIMP-2 could inhibit HCT 116 cells invasion and migration by regulating MMP-9. In sum, a combined expression of TIMP-2 and MMP-9 as efficient prognostic indicators was found for the first time.

## Introduction

Colorectal cancer (CRC), one of the most frequent cancers in human beings, is one of the common causes of morbidity and mortality [1]. It is currently estimated that there are over one-million new incident cases of CRC worldwide every year [2]. It represents the third place in male cancer mortality and the second place in female cancer mortality [3]. Despite the fact that multi-model treatment strategies are used in clinic, including surgery, chemotherapy, adjuvant radiotherapy, targeted therapy, gene therapy, traditional Chinese medicine, and other treatments, the 5-year overall survival rate of metastatic CRC still remains approximately 10% [4]. Therefore, it is urgent to discover the molecular and cellular processes involved in CRC mechanisms to develop reliable biomarkers that could predict the prognosis in CRC patients with adverse outcomes.

Tissue inhibitors of metalloproteinases (TIMPs) are endogenous proteins which could prohibit cell proliferation and migration by inhibiting the function of Matrix metalloproteinases (MMPs). Previous studies have shown that when the balance between TIMPs and MMPs was disrupted, it could lead to the degradation of the extracellular matrix and induce tumor cell invasion, migration or other receptor-mediated changes [5]. Tissue inhibitor of metalloproteinase-2 (TIMP-2) is a unique inhibitor among the TIMP family members, because it not only correlates with matrix remodeling and angiogenesis suppressing but also involves in the process of tumor growth, inflammation, and other diseases [6]. TIMP-2 predicted better prognosis in pancreatic carcinoma [7] and endometrial carcinoma [8], but poorer outcome in hepatocellular cancer [9], neuroblastoma [10], gastric cancer [11], canine mammary cancer [12], laryngeal cancer [13], and so on.

MMPs, synthesized by neoplastic and stromal cells, are mainly divided into five main categories: collagenases, matrilysins, gelatinases, stromelysins, and membrane-type metalloproteinases/membrane-type MMPs [14]. They are zinc-dependent proteases [15,16], which not only play a pivotal role in extracellular matrix (ECM) remodeling, but also closely related to the regulation of multiple stages of cancer progression [17]. Matrix metalloproteinase-9 (MMP-9) has been implicated in the progression and metastasis of various cancers, such as cervical cancer [18], and ovarian carcinoma [19]. Reconstruction of normal and tumor tissues may be caused by the imbalance between MMPs and their natural inhibitors, TIMPs [7,20–22]. Previous studies have indicated that TIMP-2 or MMP-9 could be associated with prognosis and clinicopathological features in CRC [23–25].

However, the co-expression of TIMP-2 and MMP-9 and its relevant mechanism in CRC are still unclear. In this paper, expression of TIMP-2 and MMP-9 in 470 CRC database was evaluated by the immunohistochemical method and we found that TIMP-2 could inhibit CRC metastasis by regulating MMP-9 in *vivo* and in *vitro*.

## Results

### Expression of TIMP-2 and MMP-9 in CRC tissues vs. adjacent normal tissues

**Figure 1.**
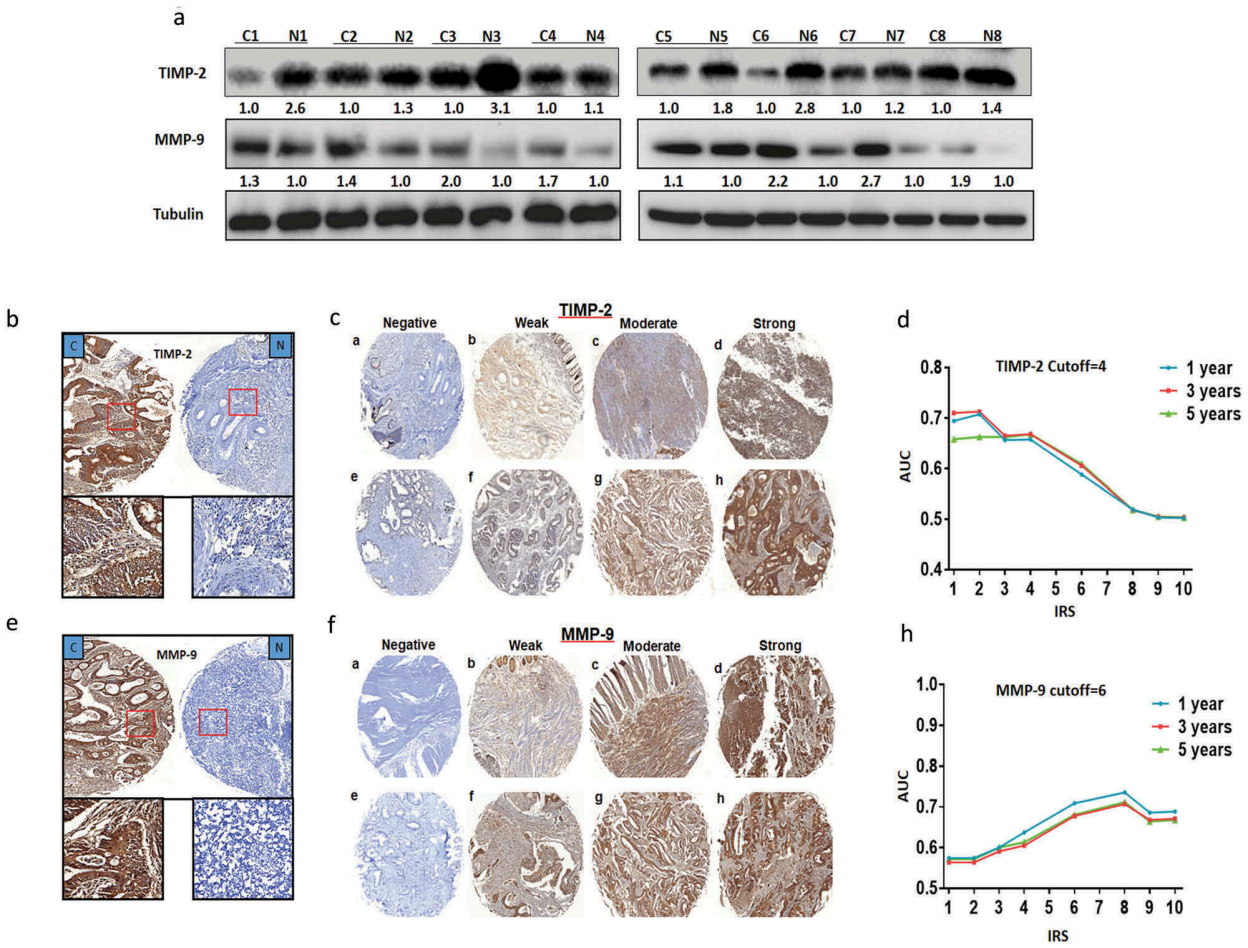
TIMP-2 or MMP-9 expression showed in CRC. (a) Expression of TIMP-2 and MMP-9 protein was detected by Western blot in cancer tissues (c) and normal tissues (N). (b, e) TIMP-2 or MMP-9 staining in CRC compared with paired normal tissues, respectively. Top panel, original magnification, 40×; bottom panel, magnification, 200× . (c, f) Representative images of TIMP-2 or MMP-9 immunohistochemical staining in TMA were showed, respectively. Note: (a-d) Adjacent normal tissue; (e-h) Cancer tissue (a, e, Negative staining. b, f, Weak staining. c, g, Moderate staining. d, h, Strong staining). All panels, original magnification, 40× . (d, h) The cut-off values of TIMP-2 or MMP-9 for immunoreactivity score (IRS) were calculated for 1, 3 and 5 years of OS time according to the area under the curve (AUC).

Eight pairs of human CRC samples, including primary CRC and matched normal colorectal tissues were collected to detect the expression of TIMP-2 and MMP-9 protein by western blotting, respectively. We found that the expression levels of TIMP-2 in tumor tissues were much lower compared within the matched normal tissues. In contrast, MMP-9 expression levels were increased (Figure 1a). Immunohistochemistry staining was utilized in TMA slides to further confirm TIMP-2 or MMP-9 expression in CRC tissues and paired adjacent non-cancerous tissues. Representative images of TIMP-2 or MMP-9 immunohistochemical staining in TMA were shown in Figure 1b and 1c and Figure 1e and 1f, respectively.

**Figure 2.**
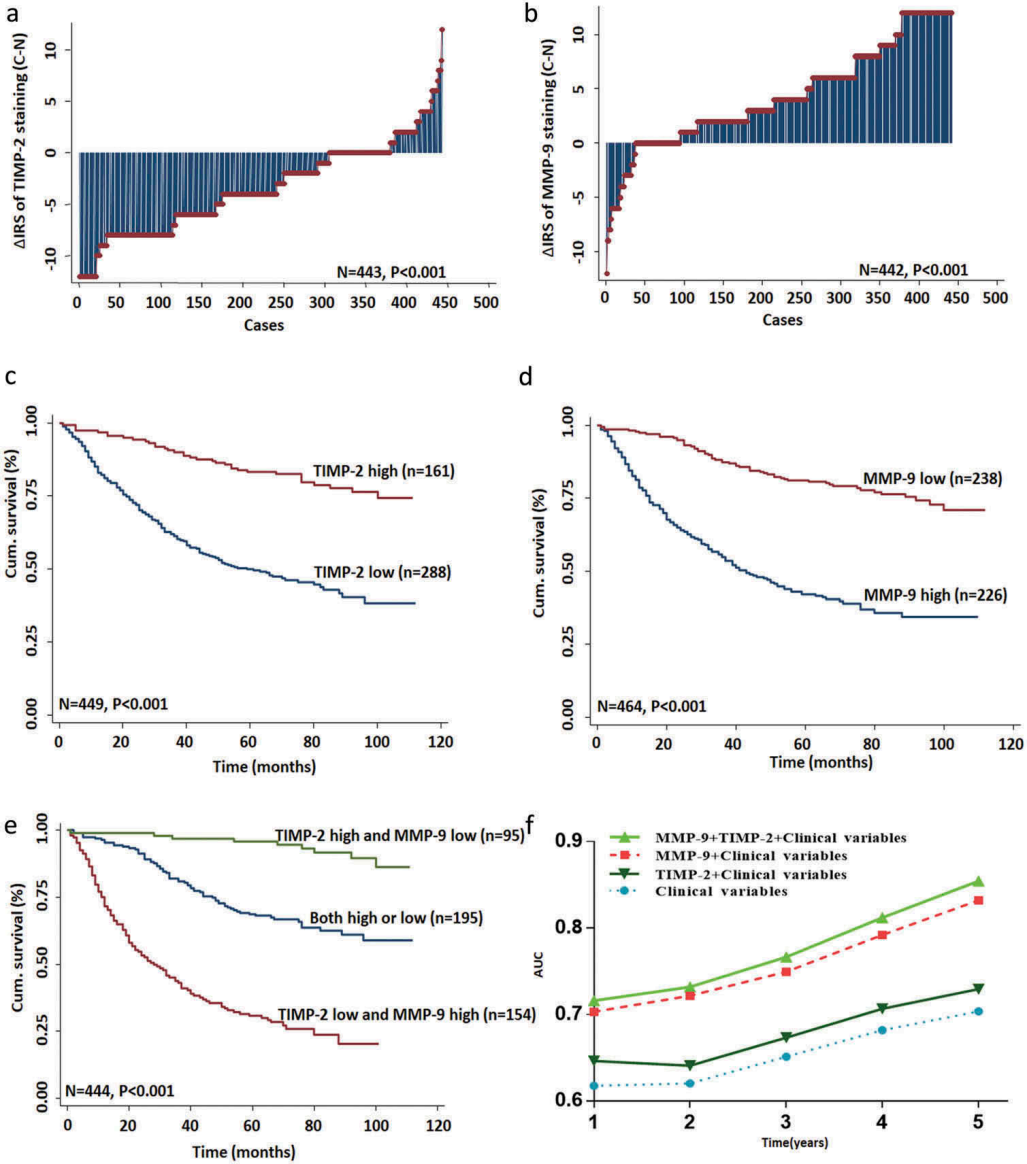
TIMP-2 or MMP-9 expression predicted prognosis of CRC. (a, b) The distribution of TIMP-2 or MMP-9 staining in TMA compared with paired normal tissues, respectively. Note: (c) cancer tissue; N, adjacent normal tissue. (c, d, e) Kaplan-Meier curves of TIMP-2, MMP-9, and combined with TIMP-2/MMP-9 expression in training cohort for OS. (f) Time-dependent ROC analyses for clinical risk score (TNM stage, histologic type, and tumor diameter), or in combination with TIMP-2, MMP-9, TIMP-2 plus MMP-9, respectively. AUC = area under the curve.

In CRC TMA slides, there were 443 cases of CRC tissues that had gained the score of cancer tissues and paired adjacent noncancer tissues. As shown in Figure 2a, we found that TIMP-2 expression was downregulated in tumor tissues compared with the paired adjacent non-tumor tissues (*P* < 0.001). Moreover, we found that MMP-9 expression was upregulated in tumor tissues compared with the paired adjacent non-tumor tissues (*P* < 0.001; Figure 2b).

### TIMP-2 or MMP-9 expression correlates with clinicopathological parameters

In the CRC cohort, Fisher’s exact analysis revealed that there was a significant positive association between low TIMP-2 expression in pathological classification (P = 0.025), depth of invasion (P = 0.003), lymph node metastasis (P < 0.001) and TNM stage (P < 0.001). However, there was no association between TIMP-2 expression and age, gender, tumor diameter and distant metastasis (Table 1).

**Table 1.** Relationship between expression levels of TIMP-2 and clinicopathological features in CRC patients a Two-sided Fisher’s exact tests b Some patients missing these clinical pathological parameters.

Variables	n = 464 cases		
	low (%)	high (%)	P^a^
All patients	288 (64.1)	161 (35.9)	
Age (years)			**0.227**
≤ 65	158 (62.5)	95 (37.5)	
＞65	130 (66.3)	66 (33.7)	
Gender			**0.17**
Males	166 (62.2)	101 (37.8)	
Females	122 (67.0)	60 (33.0)	
Pathological classification^b^			**0.025**
Ⅰ	2 (40.0)	3 (60.0)	
Ⅱ	254 (62.9)	150 (37.1)	
Ⅲ	28(82.4)	6(17.6)	
Depth of invasion^b^			**0.003**
T1/T2	49 (51.6)	46 (48.4)	
T3/T4	236 (67.6)	113 (32.4)	
Lymph node metastasis^b^			**<0.001**
N0	126 (48.3)	135 (51.7)	
N1/N2	160 (87.0)	24 (13.0)	
TNM stage^b^			**<0.001**
Ⅰ	38 (46.3)	44 (53.7)	
Ⅱ	80 (47.1)	90 (52.9)	
Ⅲ	152 (87.4)	22 (12.6)	
Ⅳ	15 (88.2)	2 (11.8)	
Tumor diameter^b^			**0.526**
≤ 5 cm	231 (64.0)	130 (36.0)	
＞5 cm	56 (64.4)	31 (35.6)	
Distant metastasis			**0.127**
M0	273 (63.5)	157 (36.5)	
M1	15 (78.9)	4 (21.1)	

We also analyzed the relationship between MMP-9 expression and clinicopathological parameters. From Table 2, data showed that MMP-9 expression in cancer tissues was significantly associated with age (P = 0.044), pathological classification (P = 0.002), depth of invasion (P < 0.001), lymph node metastasis (P < 0.001), distant metastasis (P < 0.001) and TNM stage (P < 0.001).

**Table 2.** Relationship between expression levels of MMP-9 and clinicopathological features in CRC patients.

Variables	n = 464 cases		
	low (%)	high (%)	P^a^
All patients	238 (51.3)	226 (48.7)	
Age (years)			**0.044**
≤65	145 (54.9)	119 (45.1)	
＞65	93 (46.5)	107 (53.5)	
Gender			**0.432**
Males	144 (51.8)	134 (48.2)	
Females	94 (50.5)	92 (49.5)	
Pathological classificationb			**0.002**
Ⅰ	5 (100.0)	0 (0.0)	
Ⅱ	219 (52.4)	199 (47.6)	
Ⅲ	11 (30.6)	25(69.4)	
Depth of invasionb			**<0.001**
T1/T2	83 (80.6)	20 (19.4)	
T3/T4	152 (42.6)	205 (57.4)	
Lymph node metastasisb			**<0.001**
N0	181 (66.5)	91 (33.5)	
N1/N2	55 (29.1)	134 (70.9)	
TNM stageb			**<0.001**
Ⅰ	73 (83.0)	15 (17.0)	
Ⅱ	106 (60.6)	69 (39.4)	
Ⅲ	54 (30.2)	125 (69.8)	
Ⅳ	1 (5.9)	16 (94.1)	
Tumor diameterb			**0.314**
≤5 cm	194 (51.9)	180 (48.1)	
＞5 cm	43 (48.3)	46 (51.7)	
Distant metastasis			**<0.001**
M0	237(53.3)	208(46.7)	
M1	1(5.3)	18(94.7)	

a Two-sided Fisher’s exact tests.

b Some patients missing these clinical pathological parameters.

### Low TIMP-2 or high MMP-9 expression correlates with the poor survival of CRC patients

Kaplan-Meier survival analysis was conducted by 5-year overall cumulative survival, which revealed that low TIMP-2 or high MMP-9 expression in cancer tissues was correlated with a worse OS in CRC patients (P < 0.001 and P < 0.001, respectively, log-rank test; Figure 2c and 2d). Besides, TIMP-2 or MMP-9 expression in cancer tissues was an independent marker for the prognosis of CRC patients by univariate and multivariate Cox regression analysis. The univariate Cox regression analysis also showed that age, pathological classification, depth of invasion, lymph node metastasis, distant metastasis, TNM stage, and TIMP-2 or MMP-9 expression were associated with OS of CRC patients (Table 3). The multivariate Cox regression analysis revealed that TIMP-2 or MMP-9 expression was an independent and unfavorable prognostic factor for CRC patients (TIMP-2: HR, 0.372, 95% CI, 0.250–0.554, P < 0.001; MMP-9: HR, 0.346, 95% CI 0.249–0.480, P < 0.001; Table 4).

**Table 3.** Univariate Cox regression analysis of TIMP-2 or MMP-9 expression and clinicopathological variables predicting survival in patients with CRC patients.

Variables	n = 470 cases	
	HR (95% CI)	P
Age (≤65 vs. > 65)	1.607 (1.215–2.126)	0.001
Gender (male vs. female)	1.013 (0.762–1.347)	0.927
Pathological classification (I/II vs. III)	2.475 (1.587–3.860)	＜0.001
Depth of invasion (T1/T2 vs. T3/T4)	3.687 (2.270–5.990)	＜0.001
Lymph node metastasis (N0 vs. N1/N2)	2.807 (2.112–3.731)	＜0.001
TNM stage (I/II vs. III/IV)	3.214 (2.407–4.291)	＜0.001
Distant metastasis(M0 vs. M1)	8.150 (4.849–13.69)	＜0.001
Tumor diameter (≤5 cm vs. >5 cm)	1.196 (0.848–1.688)	0.307
TIMP-2 expression (low vs. high)	0.689 (0.517–0.919)	**＜0.001**
MMP-9 expression (low vs. high)	0.639 (0.458–0.893)	**＜0.001**

**Table 4.** Multivariate Cox regression analysis of TIMP-2, MMP-9, TIMP-2/MMP-9 expression and clinicopathological variables predicting survival in patients with CRC.

Variables	HR (95% CI)	P^a^
**TIMP-2**		
Gender (male vs. female)	0.932 (0.696–1.247)	**0.634**
Pathological classification (I/II vs. III)	1.818 (1.134–2.914)	**0.013**
TNM stage (I/II vs. III/IV)	2.307 (1.684–3.160)	**＜0.001**
Tumor diameter (≤5 cm vs. >5 cm)	1.066 (0.741–1.533)	**0.731**
TIMP-2 expression (low vs. high)	0.372 (0.250–0.554)	**＜0.001**
**MMP-9**		
Gender (male vs. female)	0.986 (0.736–1.321)	**0.926**
Pathological classification (I/II vs. III)	1.890 (1.175–3.040)	**0.009**
TNM stage (I/II vs. III/IV)	2.260 (1.656–3.085)	**＜0.001**
Tumor diameter (≤5 cm vs. >5 cm)	1.063 (0.733–1.540)	**0.747**
MMP-9 expression (low vs. high)	0.346 (0.249–0.480)	**＜0.001**
**TIMP-2/MMP-9**		
Gender (male vs. female)	0.898 (0.672–1.199)	**0.465**
Pathological classification (I/II vs. III)	1.881 (1.180–2.997)	**0.008**
TNM stage (I/II vs. III/IV)	3.024 (2.238–4.085)	**＜0.001**
Tumor diameter (≤5 cm vs. >5 cm)	1.111 (0.776–1.591)	**0.564**
**TIMP-2/MMP-9 expression**		
both low vs. one low	0.106 (0.048–0.234)	**＜0.001**
both low vs. both high	0.428 (0.299–0.613)	**＜0.001**

^a^Multivariate Cox regression analysis including gender, pathological classification, TNM stage, tumor diameter, TIMP-2 or MMP-9 or combined 2 proteins expression status.

### Synergistic effect of TIMP-2 with MMP-9 expression on OS in CRC patients

Using Kaplan-Meier survival assay to assess the combined effect of TIMP-2 and MMP-9, we detected that patients with high expression of TIMP-2 and low expression of MMP-9 had a more favorable outcome of survival when compared with TIMP-2 low and MMP-9 high expression group or both high/low expression group (P < 0.001; log-rank test; Figure 2e). The multivariate Cox regression analysis indicated that high TIMP-2 and low MMP-9 expression alone was a favorable independent prognostic factor for CRC patients (P < 0.05 for all; Table 4).

To further evaluate whether TIMP-2 combined with MMP-9 has a synergistic effect on the prognosis of CRC patient, we conducted a time-dependent ROC analysis for the censored data. Our data indicated that combination of the clinical risk scores (TNM stage, histologic type, and tumor diameter), TIMP-2 or MMP-9 or TIMP-2 plus MMP-9 expression predicted prognosis of CRC. We included that the clinical risk scores with TIMP-2 plus MMP-9 expression contributed much more than any one of these markers alone in CRC patients (Figure 2f). For instance, in the TMA cohort, the AUC at year 5 was 0.703 (95% CI, 0.476–0.703) for only clinical risk score, whereas it was increased to 0.854 (95% CI, 0.665–1.026) when combined with the clinical risk score and with TIMP-2 plus MMP-9 risk score.

### TIMP-2 inhibits HCT-116 cells invasion and migration via regulating MMP-9

**Figure 3.**
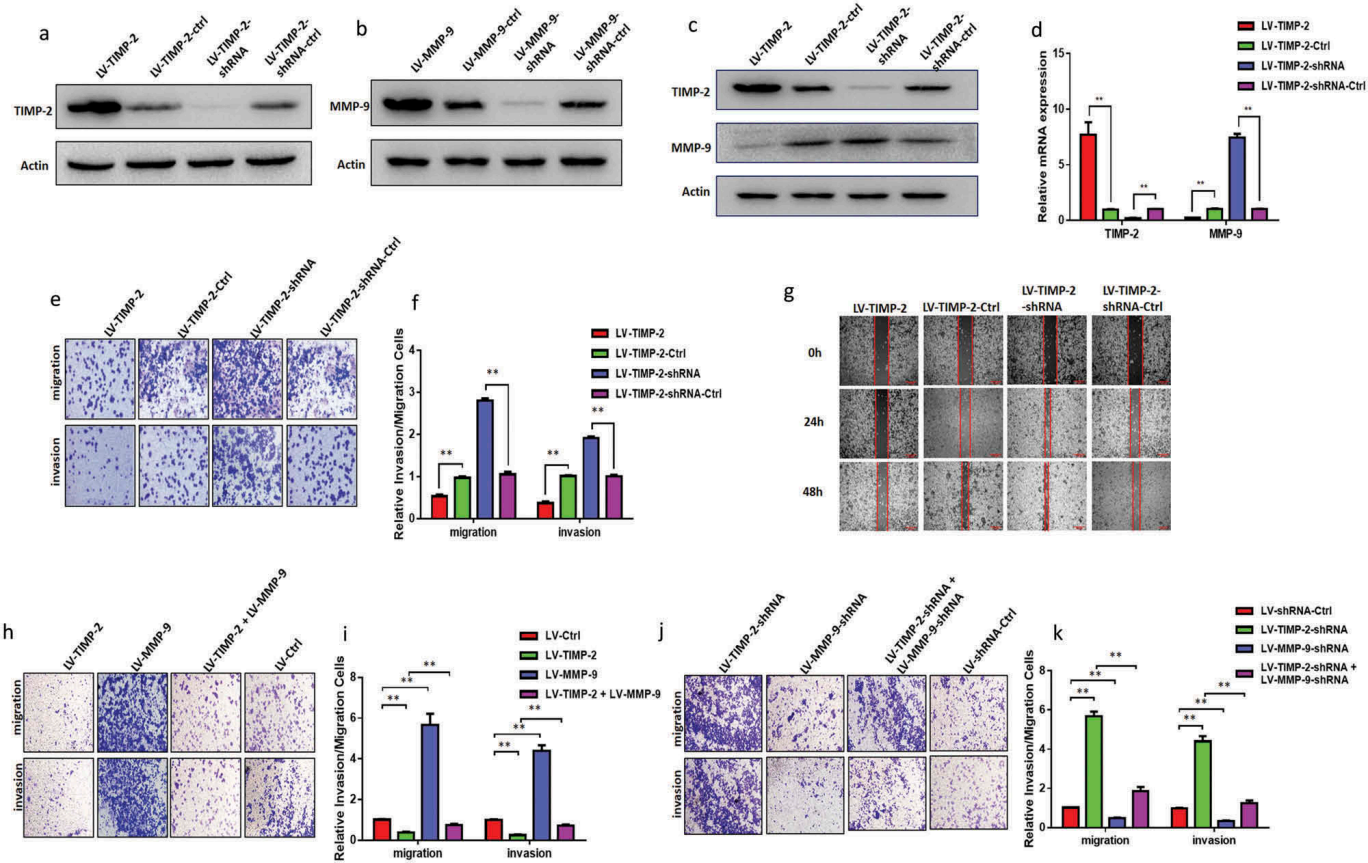
TIMP-2 inhibited HCT-116 cells migration and invasion via regulating MMP-9 in *vitro*. (a, b) Western blot was used to validate the expression of TIMP-2 or MMP-9 by virus transfection. (c) TIMP-2 protein could negatively regulate MMP-9 protein by Western blot; (d) TIMP-2 mRNA could negatively regulate MMP-9 mRNA by RT-PCR. (e, f) TIMP-2 could promote HCT-116 cells migration and invasion by transwell assay. Numbers of cell migration and invasion per field were counted in five random fields for TIMP-2 over-expressing/knockdown and control groups (n = 3/group). * P < 0.05, ** P < 0.01. (g) The migratory ability of HCT-116 cells with different TIMP-2 expression levels was detected by wound healing assay. (h, i) LV-TIMP-2, LV-MMP-9, LV-TIMP-2+ LV-MMP-9, LV-ctrl HCT-116 cells were assessed the ability of invasion and migration after 24 h incubation by transwell assay. (j, k) LV-TIMP-2-shRNA, LV-MMP-9-shRNA, LV-TIMP-2-shRNA+LV-MMP-9-shRNA, LV-shRNA-ctrl CT-116 cells were assessed after 24 h incubation by transwell assay. Note: (i, k) represent numbers of cell migration and invasion per field (n = 3/group), respectively. * P < 0.05, ** P < 0.01. E, H, J cresyl violet staining (200× magnification).

The lentivirus-mediated overexpression or knockdown of TIMP-2 or MMP-9 was analyzed by western blot (Figure 3a and 3b). As showed in Figure 3c and 3d, we found that TIMP-2 could regulate MMP-9 both on the transcriptional and translational levels. Our data indicated that the invasion and migration of LV-TIMP-2 HCT-116 cells were significantly weakened, but LV-TIMP-2-shRNA HCT-116 cells were significantly increased, compared with the corresponding control group, respectively (Figure 3e and 3f, * P < 0.05, ** P < 0.01). We also used wound healing assay to observe these 116 cells' migration capacity. As showed in Figure 3g, the migration capacity of LV-TIMP-2-shRNA HCT-116 cells was higher than the control group, but LV-TIMP-2 HCT-116 cells were lower. Through these data, we could draw a conclusion that TIMP-2 could inhibit HCT-116 cells invasion and migration.

To prove that TIMP-2 could inhibit HCT-116 cells invasion and migration by regulating MMP-9, we had a secondary lentivirus-infection to vary MMP-9 expression. We had confirmed that TIMP-2 high expression could reduce the capability of invasion and migration. After we re-infected LV-TIMP-2 HCT-116 cells with viruses to increase MMP-9 expression, the capability of invasion and migration could be increased (Figure 3h and 3I, * P < 0.05, ** P < 0.01). Using the same method, the invasion and migration capability of LV-TIMP-2-shRNA HCT-116 cells could be reduced after infection with LV-MMP-9-shRNA viruses (Figure 3j and 3k, * P < 0.05, ** P < 0.01).

### TIMP-2 inhibits angiogenesis

**Figure 4.**
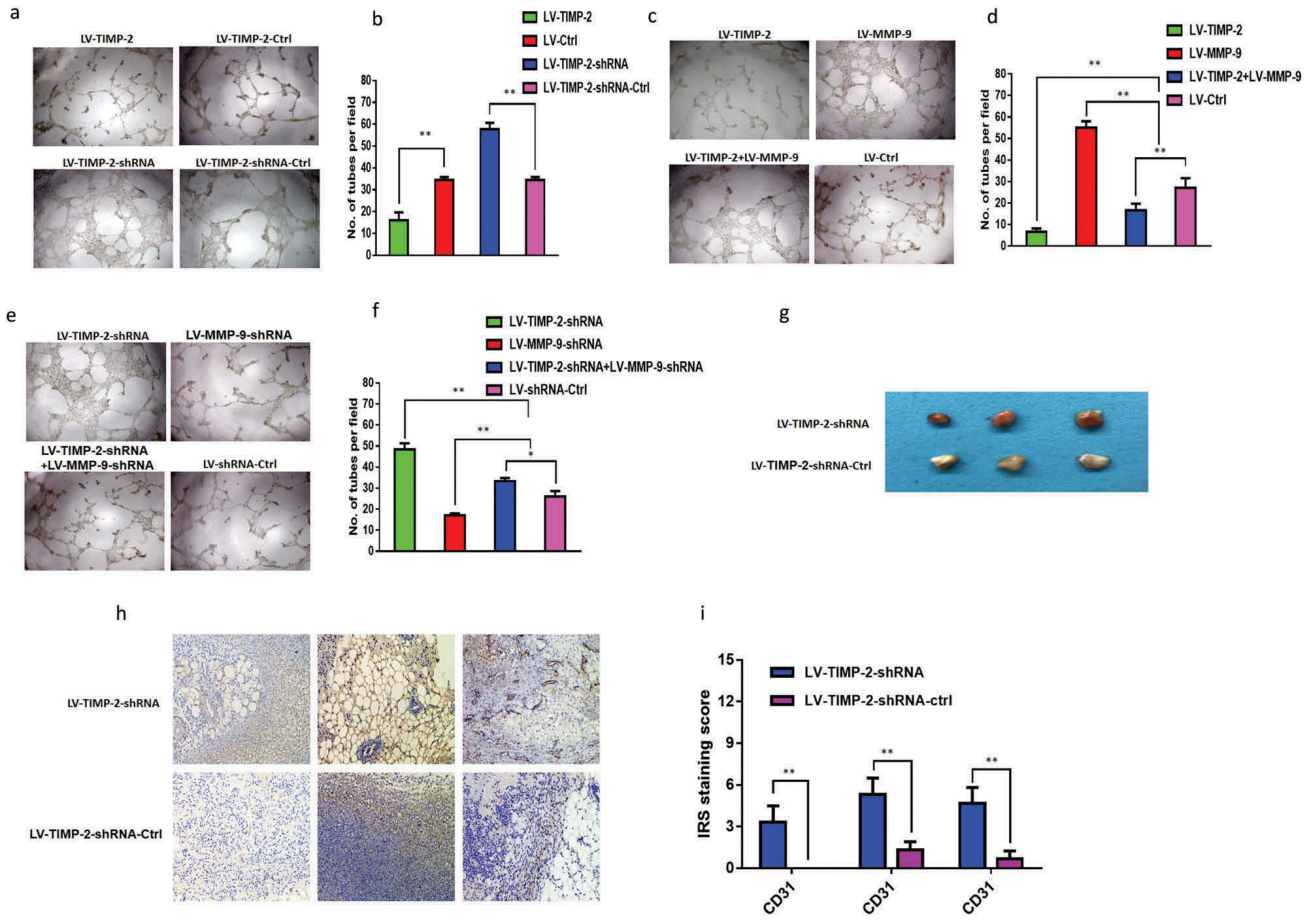
TIMP-2 inhibited angiogenesis. (a, b) Supernatant of LV-TIMP-2, LV-TIMP-2-ctrl, LV-TIMP-2-shRNA, LV-shRNA-ctrl HCT-116 cells acted on vascular endothelial cell, respectively. (c, d) Supernatant of LV-TIMP-2, LV-MMP-9, LV-TIMP-2+ LV-MMP-9, LV-ctrl HCT-116 cells acted on vascular endothelial cell, respectively. (e, f) Supernatant of LV-TIMP-2-shRNA, LV-MMP-9-shRNA, LV-TIMP-2-shRNA+LV-MMP-9-shRNA, LV-shRNA-ctrl HCT-116 cells acted on vascular endothelial cell, respectively. Note: Numbers of tubule per field were counted in five random fields in every groups (n = 3/group), respectively. * P < 0.05, ** P < 0.01. Original magnification, 40× . (g, h, i) Representative matrigel plugs in mice were photographed. Note: (g) New vasculatures in the matrigel plugs in LV-TIMP-2-shRNA or LV-shRNA-ctrl group. (h) Speciﬁc anti-CD31 antibody for blood vessels was stained by immunohistochemical method. (i) IRS of CD31 was obtained.

To confirm that TIMP-2 could inhibit angiogenesis, we used the HUVEC cells tubular formation assay to observe the numbers of tubular. We used the above supernatants of LV-TIMP-2, LV-TIMP-2-ctrl, LV-TIMP-2-shRNA, LV-TIMP-2-shRNA-ctrl HCT-116 cells to act on HUVEC cells. For 24 h treatment, we observed the numbers of tubular (Figure 4a and 4b, * P < 0.05, ** P < 0.01). From these results, we found that the supernatants of LV-TIMP-2 HCT-116 cells could inhibit tubular generation much more effectively than the control group. In contrast, the supernatants of LV-TIMP-2-shRNA HCT-116 cells could promote tubular generation.

To further confirm our observations, we used LV-MMP-9 virus to infect LV-TIMP-2 HCT-116 cells. Results showed that supernatants of LV-TIMP-2 HCT-116 cells could increase numbers of tubular after infection with LV-MMP-9 virus (Figure 4c and 4d, * P < 0.05, ** P < 0.01). In addition, the supernatants of LV-TIMP-2-shRNA HCT-116 cells could reduce numbers of tubular after infection with LV-MMP-9-shRNA virus (Figure 4e and 4f, * P < 0.05, ** P < 0.01).

We also used matrigel plug assay to observe angiogenic morphology *in vivo*. The study revealed that the matrigel plug showed no angiogenesis and was white in the control group, and in the group LV-TIMP-2-shRNA HCT-116 cells, a large amount of angiogenesis were observed in the matrigel plug (Figure 4g). The anti-CD3-related angiogenesis had been detected by immunohistochemical method and IRS of CD31 was gained (Figure 4h and 4i, * P < 0.05, ** P < 0.01).

## Discussion

CRC, as one of the common malignant tumors, its pathogenesis is a complex process that is tightly related to the abnormal expression of oncogenes and tumor suppressor genes [26]. Once this process develops abnormally, the tumor is likely to deteriorate further and lead to metastasis. About 20–25% of CRC patients present with metastatic disease at the time of diagnosis and almost 50% of CRC patients will develop metastasis [1,27]. Therefore, it is urgent to identify potential biomarkers for CRC prognosis.

TIMPs are a family of metalloproteinase tissue inhibitors, in which TIMP-2 is a typical representative. TIMP-2 is originally found to prohibit cell proliferation and migration in *vitro* via inhibiting the function of MMPs. Extensive studies revealed that TIMP-2 possessed the potential as an anticancer agent. The expression of TIMP-2 could affect in many cancers, such as lung [28], breast [29], ovarian [30], bladder [31], and cervical cancer [32]. In this study, we provided new evidences that TIMP-2 expression in CRC tumor tissues was lower than that in matched adjacent normal tissues. We also demonstrated that low TIMP-2 expression in CRC tumor tissues was closely correlated with pathological classification, depth of invasion, lymph node metastasis and TNM stage. Besides, Kaplan-Meier survival analysis revealed that the low expression of TIMP-2 in tumor tissues was associated with poor OS in CRC patients. Furthermore, univariate and multivariate Cox proportional hazards regression analysis showed that TIMP-2 expression was an independent negative prognostic factor of CRC.

MMPs play an important role in invasion and metastasis of tumor cells by affecting synthesis and degradation of ECM. Among the MMP members, the MMP-9 has been extensively studied in human cancers and has been shown to be closely related to the invasive potential and metastasis of different types of tumor cells. The MMP-9 gene is located on chromosome 20q11.2-q13.1, which contains 13 exons. It is an enzyme, which is also known as 92 kDa gelatinase, 92 kDa type IV collagenase [33,34]. In addition, MMP-9 expression was upregulated in tumor tissues compared with that noted in the paired adjacent non-tumor tissues in both CRC fresh tissues and a TMA cohort. We demonstrated that high MMP-9 expression in CRC tumor tissues was significantly related to age, pathological classification, depth of invasion, lymph node metastasis, distant metastasis, and TNM stage. The high expression of MMP-9 was also correlated with worse OS. It was found to be an independent negative prognostic factor in CRC patients. Our cohort indicated that decreased expression of TIMP-2 and increased expression of MMP-9 were significantly associated with unfavorable clinicopathologic parameters and worse OS for CRC.

Invasion and migration are the main biological characteristics associated with tumor malignancy [35,36]. MMPs and inhibitors of TIMPs play a vital role in the process of degradation of the ECM and basal membrane (BM) which is closely related to tumor invasiveness [37,38]. In our study, we had confirmed that TIMP-2 could regulate MMP-9 whether at protein or mRNA levels. Using Transwell and tubular formation assays, our data indicated that TIMP-2 inhibited invasion and migration of HCT-116 cells by regulating MMP-9. Interestingly, we also found that TIMP-2 combined with MMP-9 has synergistic potential and may be more effective than TIMP-2 or MMP-9 alone in predicting the prognosis of CRC patients.

In conclusion, our findings indicated that TIMP-2 or MMP-9 are prognostic molecular biomarkers for CRC patients. With comprehensive bioinformatics analysis of database, we currently could confirm that TIMP-2 might play an important role in the prognosis of CRC through directly affecting cell invasion, migration, and angiogenesis. To the best of our knowledge, we first revealed the combined value of TIMP-2 and MMP-9 as efficient prognostic factors which could be used for the detection of metastatic CRC. However, some limitations still involved in the study and further investigation is warranted to elucidate their role in the occurrence and development of CRC.

## Materials and methods

### Patient specimens and tissue samples

This study was granted by Institutional Review Board of Yixing Hospital affiliated to Yangzhou University Medical College. All subjects provided their written informed consent and were assured of their anonymity and the confidentiality of the data obtained. The Ethics Committee of the Yixing Hospital approved this study, which was performed according to the principles of the Declaration of Helsinki.

The eight-paired fresh samples in our study were frozen in liquid nitrogen immediately after surgical excision and kept at −80℃ until they were used for western blot analysis. Tissue microarray (TMA) was a method for collecting small disc tissues from a series of standard paraffin tissue blocks and placing them in an array of receptor paraffin blocks [39]. The cohort TMA contained 470 CRC surgical cases, which was obtained from Yixing Hospital, in the South of Jiangsu Province between 2006.01 and 2010.12. These patients were followed up at least 5 years. Overall survival (OS) was the primary endpoint of this analysis, and the survival time was calculated from the date of surgery to the date of death or to the final follow-up. The main clinical and pathologic variables of the patients were obtained. As in Table 5, the clinicopathological features contained gender, differentation stage, depth of invasion, lymph node metastasis and so on.

**Table 5.** The patients’ clinicopathologic information in CRC.

Variables	n	
All patients	470	(%)
Age (years)		
≤65	267	56.8
＞65	203	43.2
Gender		
Males	281	59.8
Females	189	40.2
Pathological classification^a^		
Ⅰ	5	1.1
Ⅱ	423	91.2
Ⅲ	36	7.7
Depth of invasion^a^		
T1	9	2.0
T2	94	20.2
T3	347	74.6
T4	15	3.2
Lymph node metastasis^a^		
N0	276	59.2
N1	126	27.0
N2	64	13.8
TNM stage^a^		
Ⅰ	88	18.9
Ⅱ	179	38.6
Ⅲ	180	38.8
Ⅳ	17	3.7
Tumor diameter^a^		
≤ 5 cm	378	80.6
＞5 cm	91	19.4
Distant metastasis		
M0	451	95.9
M1	19	4.1

^a^ Some patients missing these clinical pathological parameters.

### Western blotting

Total cellular proteins were extracted from tumor tissues and the protein concentrations were determinate by the bicinchoninic acid method. Proteins (80 μg per hole) were separated on 10% SDS-PAGE gels. The protein was electrotransferred to a polyvinylidene fluoride (PVDF) membrane. The PVDF membrane was blocked at room temperature for 2 h with Tris-buffered Saline and Tween-20 (TBS-T) containing 5% skimmed milk. After incubating in the rabbit anti-TIMP-2 (1∶1000; Epitomics, California, USA) or anti-MMP-9 (1∶1000; Cell Signaling Technology, MA, USA) at 4℃ overnight, the membranes were washed for three times with 1× TBS-T to remove excess antibodies. Secondary antibody–monoclonal anti-β-actin antibody (1∶2000; Beyotime Biotechnology, Nantong, China) was used to incubate the membranes for 2 h at room temperature, then washed with 1× TBS-T. ECL detection was used to visualize the proteins and band intensity was quantified using the software Image J software (version 1.44, Wayne Rasband, National Institutes of Health, USA) and expressed as relative intensity compared with the control.

### Quantitative real-time PCR

The total RNAs of CRC cells and FHC cells were extracted using RNeasy Mini Kit (Invitrogen, Carlsbad, USA) according to instruction of manufacturer’s manual under RNase-free condition. The purified RNAs were reversely transcribed to first-strand cDNAs by a RevertAid RT reverse transcription kit (Thermo Fisher Scientific, Waltham, MA, USA). The transcriptional expression of TIMP-2 was then subjected to SYBR Green I-based real-time quantitative PCR analysis using an Applied Biosystems 7500 Real-time PCR System (Roche Applied Science, Penzberg, Upper Bavaria, Germany).

The human TIMP-2, MMP-9, and GAPDH-specific primers (TIMP-2-F 5ʹ-ATC AGG GCC AAA GDG GTC AGT G-3ʹ and TIMP-2-R, 5ʹ- GTC ACA GAG GGT GAT GTG CAT- 3ʹ; MMP-9-F 5ʹ- CGG AGA CGG GGG AGC TGG ATA ATG-3ʹ and MMP-9-R 5ʹ- GCG CGG CAG GTC TTC GGA GTA GTT – 3ʹ; GAPDH-F, 5ʹ-ACG GAT TTG GTC GTA TTG GG-3ʹ and GAPDH-R, 5ʹ-CGC TCC TGG AAG ATG GTG AT-3ʹ) (Sangon Biotechnology Inc., Shanghai, China) were used. The relative expression level of TIMP-2, MMP-9 mRNA was normalized to an internal control GAPDH and analyzed by using the 2^−ΔΔCt^ method. All reactions were performed in duplicate.

### Construction of tissue microarray (TMA) and immunohistochemistry

The appropriate formalin-fixed paraffin-embedded (FFPE) tissue specimens and adjacent normal tissues were chosen based on clinical data and used for TMA construction. The CRC TMA contained 940 cores. Each sample was punched to 1.5 mm diameter. The standard protocol for the immunostaining is provided as a previous study [40]. Rabbit monoclonal antibodies against TIMP-2 (1∶200, Epitomics, California, USA) and MMP-9 (1∶200, Cell. Signaling Technology, MA, USA) were incubated at 4℃ overnight. The omission of primary antibody was used as a negative control. As a quality control for immunostaining, the staining fraction of the tissue controlled in each microarray slide was pre-evaluated.

### Evaluation of immunostaining

The staining of TIMP-2 or MMP-9 in the tissue individual spot was scored by two pathologists. The semi-quantitative immunoreactivity score (IRS) was applied in the training cohort, as reported elsewhere [41]. The optimum cutoff value of IRS was obtained by receiver operator characteristic (ROC) analysis, and the area under the curve (AUC) at different cutoff values of the TIMP-2 IRS for 1, 3, and 5 years of overall survival time was calculated. The optimum value of cutoff points of the TIMP-2 IRS was showed to be 4 since it had the best predictive value for survival (Figure 1d). Under these conditions, samples with IRS 0–3 and IRS 4–12 were classified as low or high expression of TIMP-2, respectively. By using the same method, the optimum cutoff points of MMP-9 IRS were showed to be 6 (Figure 1h), samples with IRS 0–5 and IRS 6–12 were classified as low or high expression of MMP-9, respectively.

### Cell lines and animals

The human HCT-116 cells (Shanghai Cell Bank of the Chinese Academy of Sciences Shanghai Institute of Cell Biology, Shanghai, China) were used. The cells were cultured with RPMI-1640, supplemented with 10% FBS and incubated at 37℃ in a regulated incubator in an atmosphere with 5% CO_2_. All the cells were used at logarithmic growth phase in the whole experiments.

Female BALB/c nude mice were obtained from the Comparative Medicine Laboratory Animal Center [License No. scxk (SU) 2012–0004] of Yangzhou University (Jiangsu, China). The mice aged 6–8 weeks were maintained in specific pathogen-free conditions and cared in accordance with the National Institutes of Health Guide for the Care and Use of Laboratory Animals. The protocols were approved by the Institutional Animal Care and Use Committee (IACUC) of Yangzhou University.

### Lentiviral infection and generation of stable cell lines

The HCT-116 cells were infected with LV-TIMP-2, LV-TIMP-2-ctrl, LV-TIMP-2-shRNA and LV-TIMP-2-shRNA-ctrl (LV-MMP-9, LV-MMP-9-ctrl, LV- MMP-9-shRNA and LV-MMP-9-shRNA-ctrl) at an MOI of 20 plus 10 μg/ml of Polybrene (GeneChem, Shanghai, China), respectively. Eight hours after lentiviral infection, the HCT-116 cells were maintained with normal RPMI-1640 culture medium. Subsequently after 24 h, the cells were selected with puromycin (Gibco-BRL, Gaithersburgh, MD, USA) at a final concentration of 2 μg/ml. The knockdown and overexpression efficiency of TIMP-2 or MMP-9 were further analyzed by Western blot.

These infected viruses belonged to the lentivirus (GeneChem, Shanghai, China), which could package the specific sequence of TIMP-2 or MMP-9 gene to increase or reduce these protein expressions as a vector. These lentivirus carried the characteristics of anti-pyromycin and green fluorescence.

### Wound healing assay

The LV-TIMP-2 and LV-TIMP-2-shRNA HCT-116 cells and the corresponding control cells (2 × 10^5^ cells) were cultured in 6-well plates and grown to 90% pore floor area. Micropipette tips were used to make linear scratches, and the exfoliated cells were washed off with PBS for three times. Cells were treated with RPMI-1640 medium containing 2% FBS. The process of tumor cell migration was observed and photographed at a low-power field (50×) under microscopy at 0, 24 and 48 h after wounding.

### Transwell invasion assay

The transwell filter inserts were coated without or with matrigel (1∶8, Becton Dickinson Labware, Bedford, MA, USA) for the cell migration and invasion assays, respectively. The solution was kept at 37℃ for 1 h to transform the matrigel aggregate into gel. Then, the HCT-116 cells (5 × 10^5^ cells/100 μl serum-free RPMI-1640) were seeded onto the upper chamber of Transwell filters (8 μm pore size, Millipore, Billerica, MA, USA). The bottom chamber was filled with 500 μl RPMI-1640 medium containing 10% FBS. After 24-h incubation, the cells on the upper surface of filters were carefully removed with a cotton swab. The cells that had traversed the membrane were fixed with 95% methanol for 15 min, stained with 0.1% crystal violet for 20 min. Images were captured and counted under a microscope at 200× magnification (Nikon Corporation, Tokyo, Japan).

### HUVEC cells tubular formation assay

The HCT-116 cells were infected with LV-TIMP-2, LV-MMP-9, LV-TIMP-2-shRNA, LV- MMP-9-shRNA and the corresponding control virus. After puromycin screening, cell supernatants were collected from the arranged group. The supernatants were transferred into 15 ml centrifuge tubes and centrifuged at 500 × g at 4℃ for 5 min. Then, the supernatants were collected for further experiments.

Fifty-microliter matrigel (BD Biosciences) was wrapped per hole of a 96-hole plate that had been incubated at 37℃ for 2 h. Human umbilical vein endothelial cells (HUVECs; American Type Culture Collection, Manassas, VA, USA) were diluted by these collected supernatants (1.0 × 10^5^ cells/ml). One hundred-microliter HUVECs suspension was added per hole, culturing for 24 h. The tubular shape was observed and counted under a microscope at 40× magnification (Nikon Corporation, Tokyo, Japan). The number of tubes in five different fields were recorded, calculating the average number. For rigor, three independent experiments were performed in triplicate.

### Matrigel plug assay in vivo

The matrigel plug assay was employed as previously described [13] with some modifications. In short, 500 µl matrigel containing 100 ng of VEGF, 20 IU of heparin with these above supernatants was subcutaneously injected into abdominal midline subcutaneous of ICR mice (n = 4 each group). Eight days later, intact matrigel plugs were carefully exposed, frozen and embedded with OCT (optimal cutting temperature) compound and sliced. To identify infiltrating endothelial cells to form the blood vessels, immunofluorescence analysis was performed with anti-CD31 monoclonal primary antibody. Experimental protocols were performed under the guidelines supplied by the National Cancer Institute. The protocol was approved by Yangzhou University.

### Statistical analysis

SPSS 20.0 software (SPSS, Inc, Chicago, IL) was used for statistical processing. Fisher’s exact test was used to evaluate the relationship between TIMP-2 or MMP-9 expression and their clinicopathological parameters. IRS for TIMP-2 or MMP-9 staining in primary tumors and their paired adjacent normal tissues were assessed by the paired Wilcoxon test (raw scores). Probability of differences in OS as a function of time was ascertained by use of the Kaplan-Meier method, with a log-rank test probe for significance. Univariate and multivariate Cox proportional hazards regression analysis was performed to estimate the crude hazard ratios (HRs), adjusted HRs and 95% CI of HRs. We evaluated the performances of different scores by plotting (t, AUC [t]) for different values of follow-up time (t). All the statistical analyses were performed by STATA statistical software (version 10.1; StataCorp, College Station, TX). *P* value of <0.05 was deemed statistically significant.
